# Quantification of Estrogen Receptor-Alpha Expression in Human Breast Carcinomas With a Miniaturized, Low-Cost Digital Microscope: A Comparison with a High-End Whole Slide-Scanner

**DOI:** 10.1371/journal.pone.0144688

**Published:** 2015-12-14

**Authors:** Oscar Holmström, Nina Linder, Mikael Lundin, Hannu Moilanen, Antti Suutala, Riku Turkki, Heikki Joensuu, Jorma Isola, Vinod Diwan, Johan Lundin

**Affiliations:** 1 Institute for Molecular Medicine Finland (FIMM), Helsinki, Finland; 2 Center of Microscopy and Nanotechnology, University of Oulu, Oulu, Finland; 3 BioMediTech, University of Tampere, Tampere, Finland; 4 Helsinki University Hospital and University of Helsinki, Helsinki, Finland; 5 Department of Public Health Sciences, Karolinska Institutet, Stockholm, Sweden; ACTREC, Tata Memorial Centre, INDIA

## Abstract

**Introduction:**

A significant barrier to medical diagnostics in low-resource environments is the lack of medical care and equipment. Here we present a low-cost, cloud-connected digital microscope for applications at the point-of-care. We evaluate the performance of the device in the digital assessment of estrogen receptor-alpha (ER) expression in breast cancer samples. Studies suggest computer-assisted analysis of tumor samples digitized with whole slide-scanners may be comparable to manual scoring, here we study whether similar results can be obtained with the device presented.

**Materials and Methods:**

A total of 170 samples of human breast carcinoma, immunostained for ER expression, were digitized with a high-end slide-scanner and the point-of-care microscope. Corresponding regions from the samples were extracted, and ER status was determined visually and digitally. Samples were classified as ER negative (<1% ER positivity) or positive, and further into weakly (1–10% positivity) and strongly positive. Interobserver agreement (Cohen’s kappa) was measured and correlation coefficients (Pearson’s product-momentum) were calculated for comparison of the methods.

**Results:**

Correlation and interobserver agreement (r = 0.98, p < 0.001, kappa = 0.84, CI95% = 0.75–0.94) were strong in the results from both devices. Concordance of the point-of-care microscope and the manual scoring was good (r = 0.94, p < 0.001, kappa = 0.71, CI95% = 0.61–0.80), and comparable to the concordance between the slide scanner and manual scoring (r = 0.93, p < 0.001, kappa = 0.69, CI95% = 0.60–0.78). Fourteen (8%) discrepant cases between manual and device-based scoring were present with the slide scanner, and 16 (9%) with the point-of-care microscope, all representing samples of low ER expression.

**Conclusions:**

Tumor ER status can be accurately quantified with a low-cost imaging device and digital image-analysis, with results comparable to conventional computer-assisted or manual scoring. This technology could potentially be expanded for other histopathological applications at the point-of-care.

## Introduction

Breast cancer is the most common form of cancer in women and a major health burden in both developing and developed countries [1]. Annually over a million new cases are diagnosed globally, and in less developed countries breast cancer is the leading cause of cancer death among women [2]. The incidence of breast cancer has been uniformly rising for the last decades in most countries and is predicted to increase especially in the populations of Africa, Asia and South America, mainly due to the increased proportion of the elderly population [3]. Due to the absence of adequate healthcare in low-resource environments, a large amount of breast cancers are still discovered at a late stage, which negatively affects prognosis [4].

Assessment of cancer estrogen receptor-alpha expression in breast carcinomas is essential in their management, as breast cancer estrogen receptor (ER) expression is a strong predictive factor for response to hormonal therapies, such as tamoxifen, and also has prognostic value [5–7]. Breast cancer ER status is traditionally assessed from immunohistochemically stained tumor sections using visual scoring [8], although this method is prone to subjectivity due to the staining reaction often being heterogeneous in intensity [9]. Methods to reduce subjectivity have been suggested, such as semiquantitative scoring formulas [10], computer-assisted techniques [11], and assessment guidelines [8]. In general, the results of manual scoring of immunostained slides are in good agreement when tumor samples are strongly positive for ER expression. With samples of lower levels of ER positivity, interobserver and intraobserver variability becomes an issue, and is especially concerning with borderline positive samples [12–14].

Recent studies suggest that computer-assisted scoring of digitalized tissue slides yields results comparable to manual scoring, and may provide a more reproducible method than visual scoring of ER expression [15–18]. Image-analysis is usually carried out with high-end whole-slide scanners to create virtual slides for computer-assisted analysis. As conventional whole-slide scanners are expensive and require trained personnel and regular maintenance, this technique is limited to well-equipped laboratories [19]. The technological advances and rapid growth of the consumer electronics market during the last decade have made the mass-production of miniaturized, optomechanical components for optical imaging devices like modern camera phones very cost-efficient. These low-cost components have been utilized in prototypes of miniature digital microscopes and other optical imaging devices for point-of-care (POC) applications [20], which show promise especially in the diagnosis of infectious diseases, such as malaria and tuberculosis [21, 22].

The objective of the present study was to evaluate the performance of a cloud-connected, novel, low-cost digital microscope with slide-scanning capabilities in the digital quantification of ER expression in human breast cancer, and to compare the results with both visual scoring and digital image-analysis of virtual slides produced by a conventional whole-slide scanner. The results suggest that ER expression can be quantified accurately with a low cost device. Importantly, this technology can potentially be expanded to analysis of other proteins and pathogens in histological samples. To our knowledge, a similar study has not been conducted earlier.

## Materials and Methods

### Samples, tissue microarrays and immunohistochemical staining

Two tumor tissue microarray (TMA) slides, representing a total of 193 samples of human breast tissue were selected for the study. All cores selected for the microarrays were verified as representative breast cancer tissue by a certified pathologist. One hundred and thirty-five samples were selected from the Finprog Breast Cancer Database (http://www.finprog.org), which includes cases from the nationwide study on women diagnosed with breast cancer in 1991–2 in five geographical regions of Finland. The formalin-fixed paraffin-embedded, tumor samples were obtained from the archives of diagnostic pathology laboratories as previously described [23]. All samples had been routinely collected for diagnostic purposes and processed following local standards of procedure. The core biopsies represents sections of 5 μm thickness, punched with a 0.6 mm needle. Immunostaining for ER was performed with the mouse monoclonal anti-ER primary antibody 6F11 (Novocastra Laboratories Ltd., Newcastle, United Kingdom; dilution 1:500), visualized with an anti-mouse-peroxidase polymer (Powervision; Immunovision Inc., Daly City, CA) and the 3–3’-diaminobenzidine (DAB) chromogen and counterstained with haematoxylin.

The remaining 58 samples were selected from the Predect-series of breast cancer tissues (www.predect.eu; predect.webmicroscope.net) an Innovative Medicine Initiative (IMI)-collaboration of 21 organizations in the European Union (partners including academic and biotech laboratories and pharmaceutical industry). These samples were immunohistochemically stained for ER using an Autostainer (Lab Vision Autostainer 480S [Thermo Fisher Scientific Inc., Waltham, USA]), the rabbit anti-ER primary monoclonal antibody ab16660 (Abcam, Cambridge, UK), visualized with an anti-rabbit-peroxidase polymer (Immunologic, Duiven, Netherlands) and DAB and counterstained with haematoxylin.

### Slide digitization

**Fig 1:**
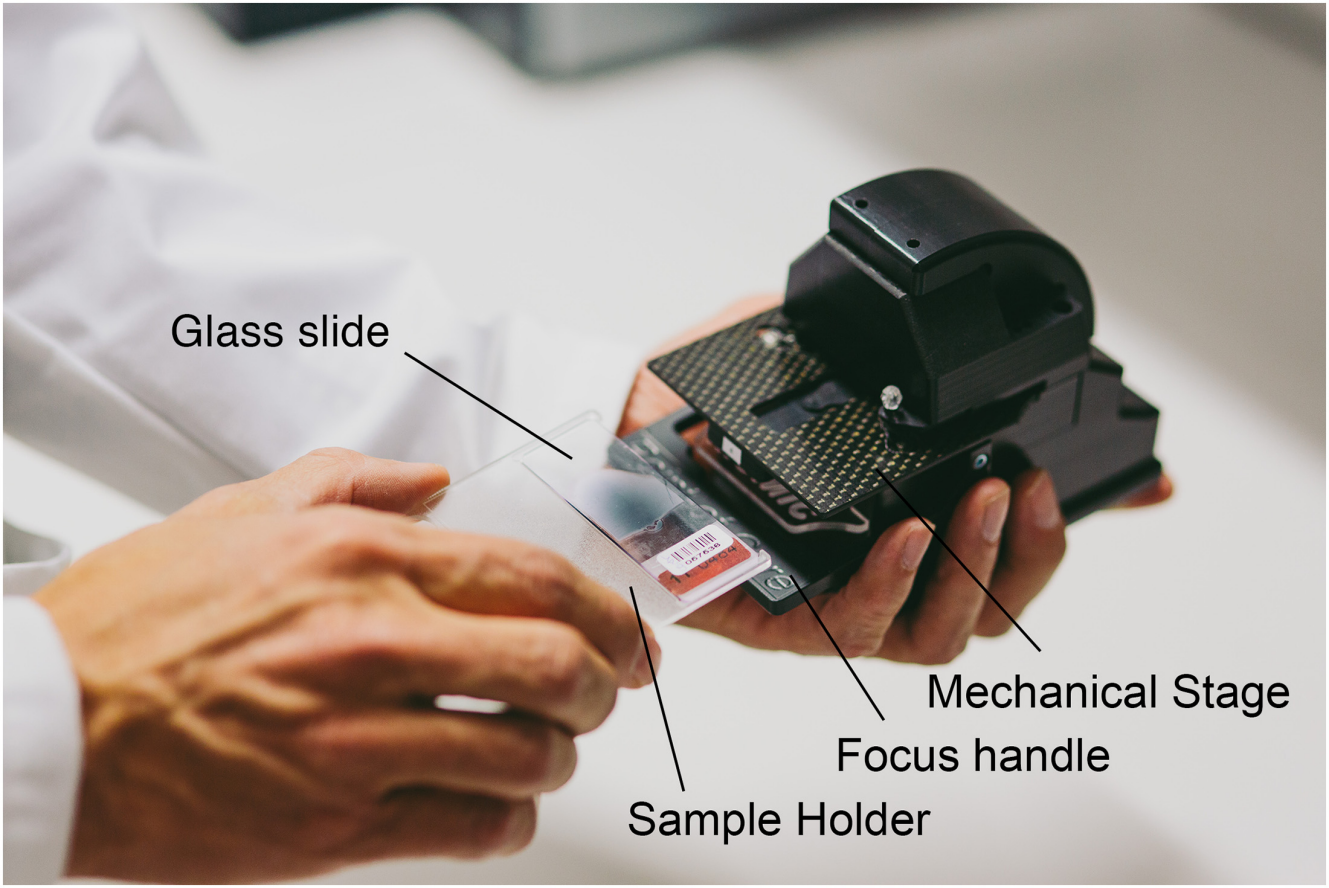
MoMic miniature microscope prototype. The glass slide is placed in the sample holder which is then inserted on the mechanical stage, and used for manual adjustment of the sample position.

The miniature microscope, “MoMic”, presented in this article represents a portable, miniature (dimensions: 115x75x115 mm) digital microscope constructed using inexpensive plastic optomechanical components, typically utilized in mobile phone camera systems (Fig 1). By reducing the tube length of the microscope to approximately a tenth of the tube length of a conventional light microscope and reversing the camera module lens, the physical size of the device could be significantly reduced.

**Fig 2:**
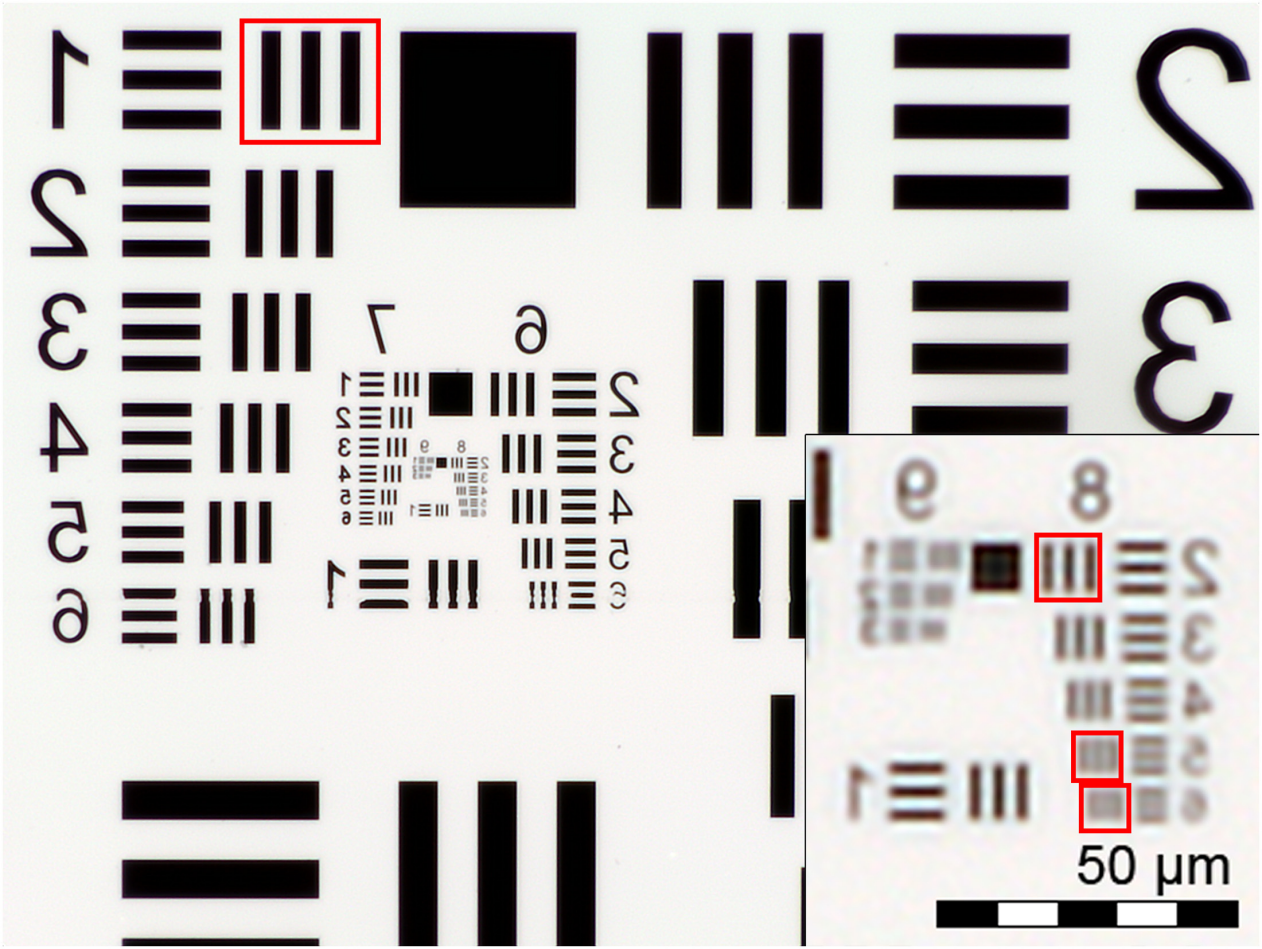
US Air Force 1951 three-bar resolution test chart. Image of standard 1951 USAF resolution target, captured with the point-of-care microscope. Due to the reversed camera lens the original image appears reversed horizontally.

**Fig 3:**
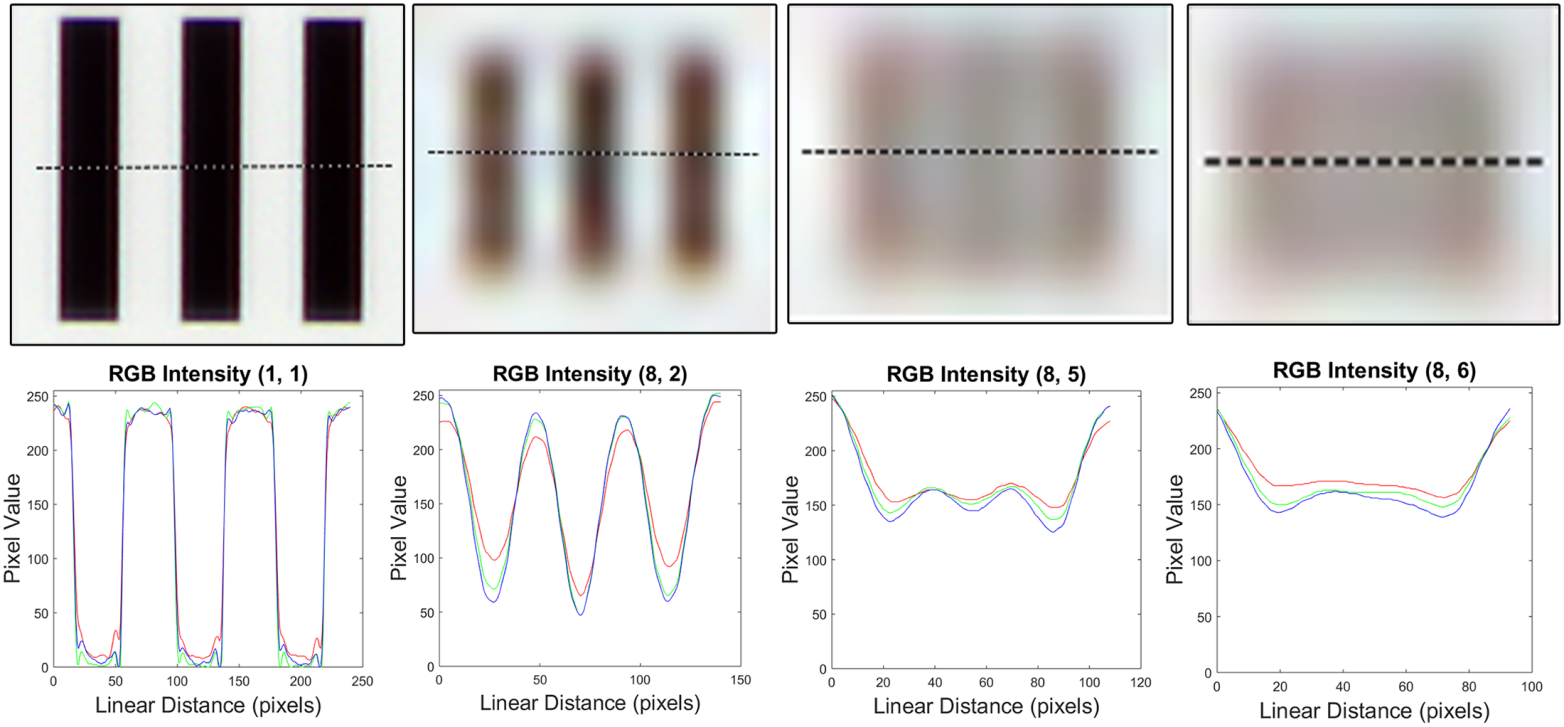
Pixel intensity profiles, calculated from four different sets of bars in the USAF three-bar resolution test chart. Intensity values (RGB) calculated from horizontal lines across regions indicated with red bounding boxes in Fig 2. From left to right: Group 1, element 1; group 8, element 2; group 8 element 5 (smallest resolvable); group 8, element 6.

A white LED irradiation modulated by a visible wavelength permeable volume diffuser, provides the source of the transmitted light. Fluorescent imaging is possible by utilizing a retractable band pass filtered UV LED. The camera module (CM6787-O500BA-E, TRULY Optoelectronics Ltd., Hong Kong) used in the microscope features a 5 MP CMOS (pixel size 1.4 μm, maximum resolution 2592x1944 pixels) image sensor and a ¼” plastic lens assembly with an effective focal length of 3.37 mm. The pixel size of the device was measured to be 0.38 μm/px with a scale bar, rendering a field of view of 0.98x0.74 mm^2^ with a total magnification of 3.7x. Coarse focus can be manually adjusted with a mechanical lever and fine focus adjusted using a voice coil actuation integrated in the camera module. Spatial resolution was determined by the smallest set of distinguishable bars in a high resolution US Air Force (USAF) three-bar resolution chart (Fig 2) and estimated to be 1.23 μm (Fig 3). The resolution is comparable to that of a conventional light microscope with a 10x objective (1.10 μm) and therefore presumed to be sufficient to resolve the objects of interest in the current study (approx. 5–10 micrometer sized stained nuclei of epithelial cells) (S3 Fig).

The digital microscope is connected to a PC by two USB2.0 ports. A 230V wall outlet power adapter provides the power source for the stage motors. As the motors run on 5V DC, they are therefore also compatible to be powered by USB ports or similar power sources (i.e. battery packs). A custom software written in the matrix laboratory (MATLAB, MathWorks Inc, Natick, MA) mathematical computing environment works both as the controller interface and the image acquisition software. Images are saved on the hard drive of the computer and uploaded to an image processing and management platform (WebMicroscope, Fimmic Oy, Helsinki, Finland) running on a cloud server (CSC—IT Center for Science Ltd, Espoo, Finland). The software features a live stream from the camera, control of brightness, focus, exposure and resolution (VGA or QSXGA). Basic image processing techniques are used to enhance the quality of captured images. These include flat field correction to correct the color balance and white light distribution caused by variations in the pixel-to-pixel sensitivity of the detector and lens shading, by using a blank field image captured without any sample. Other options include contrast enhancement (utilizing histogram stretching by simple linear interpolation between the darkest and brightest values), median filtering (3-by-3 neighborhood technique) and sharpening. Also featured is an option for high dynamic range (HDR) imaging which combines multiple images taken at different exposure levels to compensate for over- and underexposed areas of the image. The optimal configuration of the software was decided by a researcher (O.H.) by visual comparison of captured images and was not changed during the study. Navigation and adjustment of the sample is possible either manually by hand or by utilizing an external motor unit. The motor is operated from the computer software and can be utilized to scan samples larger than a single field-of-view by automatically navigating the sample, while the device captures multiple images. The images obtained in this study were saved in the Tagged Image File Format (Adobe Systems Inc., San Jose, California, USA) and stitched to virtual TMA slides, corresponding to the virtual slides obtained with a conventional slide scanner. The virtual slides were compressed to a wavelet file format (Enhanced Compressed Wavelet, ECW, ER Mapper, Intergraph, Atlanta, Georgia) with a compression ratio of 1:9 and uploaded to a whole-slide image (WSI) management server (WebMicroscope, Fimmic Oy, Helsinki Finland) running an image server software (WebMicroscope, Fimmic Oy, Helsinki, Finland). This level of compression preserves a level of spatial detail sufficiently to not alter results significantly, as shown in earlier work [24]. The corresponding digital slides can be accessed remotely with a browser or via image analysis tools (e.g. ImageJ and MATLAB).

The reference whole-slide scanner used in the study (Pannoramic 250 FLASH, 3DHISTECH Ltd., Budapest, Hungary) uses a Plan-Apochromat 20x objective (Numerical Aperture 0.8) and a VCC-F52U25CL camera (CIS, Tokyo, Japan) featuring three 1,224x1,624 pixel Charge Coupled Device (3CCD) sensors. The pixel size of the sensors is 4.4x4.4μm, yielding an image resolution of 0.22μm/pixel with the 20x objective and 1.0 adapter. Captured digital slides scanned with the whole-slide scanner were compressed to the wavelet file format and uploaded to the WSI management server using the parameters described above.

### Manual annotation

**Fig 4:**
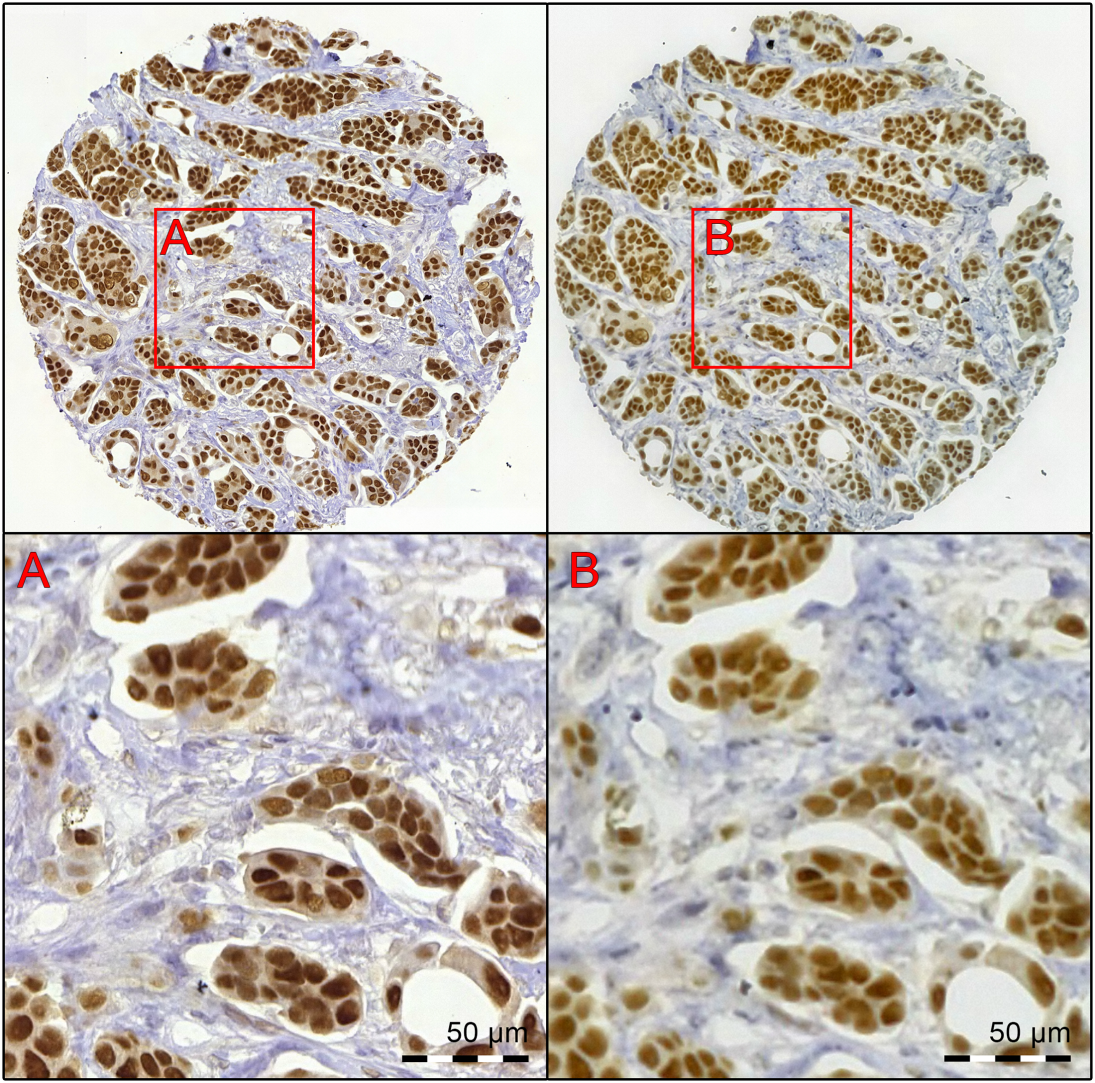
A breast cancer core biopsy, immunostained for estrogen receptor-alpha digitized with both imaging devices. The virtual sample has been digitized with the reference slide-scanner (left) and the point-of-care microscope (right). Regions marked with red bounding boxes represent tiles extracted for analysis. These can be seen at higher magnification in the lower part of the image (A. Reference slide-scanner B. Point-of-care microscope).

Manual annotation of the digitalized samples was decided to be used as the reference in the study. This was achieved by visually classifying all visible cells as either ER-positive or negative to obtain a numerical value for cancer ER-positivity (the percentage of positively staining cells). To accomplish this, smaller regions of a fixed size were extracted from the corresponding virtual slides using each device. The miniature microscope slide and the reference whole-slide scanner virtual slide were opened in the WSI slide management software environment, and conjugate points were marked by visual approximation on the images. Using the coordinates of these corresponding points, quadratic regions of a fixed size (approx. 0.05 mm^2^) were extracted, yielding 193 matching pairs of images (one image from the whole-slide scanner virtual slide and one from the MoMic virtual slide). The corresponding images were saved in the Portable Network Graphics (PNG) format using lossless compression. The pairs of images were registered based on their intensity values and further cropped using a program written in MATLAB to represent the same tumor areas as exactly as possible (Fig 4). Thirteen samples were excluded in this phase, as matching tumor regions could not be reliably registered by the software. This was likely caused by images with regions out of focus or otherwise lacking visual landmarks. Due to the higher resolution of the images from the slide-scanner (901 x 901 pixels), the images from the MoMic microscope were scaled up from the original resolution (600 x 600 pixels) to the same resolution.

As the images extracted represented exactly the same areas of the tumors, only the images taken with the whole-slide scanner were annotated. The reason for selecting the whole-slide scanner over MoMic was the higher spatial resolution of the device, thus potentially making the annotation easier and more accurate. The annotation was performed by a researcher (O.H.) with a software written in MATLAB, which provided a tool for manually marking cells in the image as positive or negative and counting the total number of positive and negative cells annotated per image [25]. The total number of cells manually annotated in the complete study set was 31 117, resulting in an average of 173 manually annotated cells per sample.

### Computer-assisted image analysis

Quantitative image-analysis of the digitalized data was performed with the commercially available software ImmunoRatio2 (Jilab Inc, Tampere, Finland) [26]. The software utilizes color separation by deconvolution, nuclear thresholding, particle segmentation and filtering to distinguish nuclei in the images, and calculates the numerical total value of positive and negative nuclei in the sample. A training series consisting of 10 representative image pairs was selected for configuration of the software. The remaining 170 pairs of images represented the test series. The configuration was done by a researcher (O.H.), and included adjusting parameters such as a staining threshold (for DAB and hematoxylin), pixel size, average nuclei size and cut-off values for rejection of small nuclei. Even though both image series represented exactly the same tumor areas with a fixed image size, different configurations of the staining thresholds had to be used for each device due to the different color profiles of the captured images. Both chromogen and counterstain thresholds were adjusted to compensate for the higher color saturation of the MoMic images. The analysis of the test series was done without human supervision, i.e. the predetermined configuration of the algorithm was not changed during the analysis. The resulting numerical cell counts and ER positivity values for each sample were entered into a spreadsheet table (Microsoft Excel, Microsoft, Redmond WA).

### Statistical analysis

Statistical analysis was performed using SPSS 22.0.0 for Windows (SPSS Inc., Chicago, USA). Presence of staining in one percent or more of tumor cells was considered the criteria for ER-positivity of the tumor [8]. Using the cut-off values of 1% and 10%, the ER positive samples were further classified as weakly positive (1–10% ER positive nuclei) and strongly positive (>10% positive nuclei). Concordance between the automated devices and the manual scoring was estimated with kappa statistics (kappa values 1–20 were considered as slight, 0.21–0.40 fair, 0.41–0.60 moderate, 0.61–0.80 good and 0.81–1.00 as high agreement [18]). Correlation coefficients were calculated with the Pearson product-moment correlation method. Bland-Altman plots were used to illustrate the agreement between the methods.

### Ethical Statement

This manuscript reports a retrospective study of routinely collected formalin fixed paraffin embedded breast cancer tissue samples. The study was approved by the Central Laboratory for the Hospital District of Helsinki and Uusimaa, HUSLAB, the Ethical Committee of Surgery of the Hospital District of Helsinki and Uusimaa (No. 94/13/03/02/2012) and The Ministry of Social Affairs and Health (No.123/08/97). According to the Ministry of Social Affairs and Health, Finland Act On the Medical Use of Human Organs, Tissues and Cells (Amendments up to 277/2013 included), written informed consent was not required because no clinical records were retrieved and the study contained no personal identifiers.

## Results

**Fig 5:**
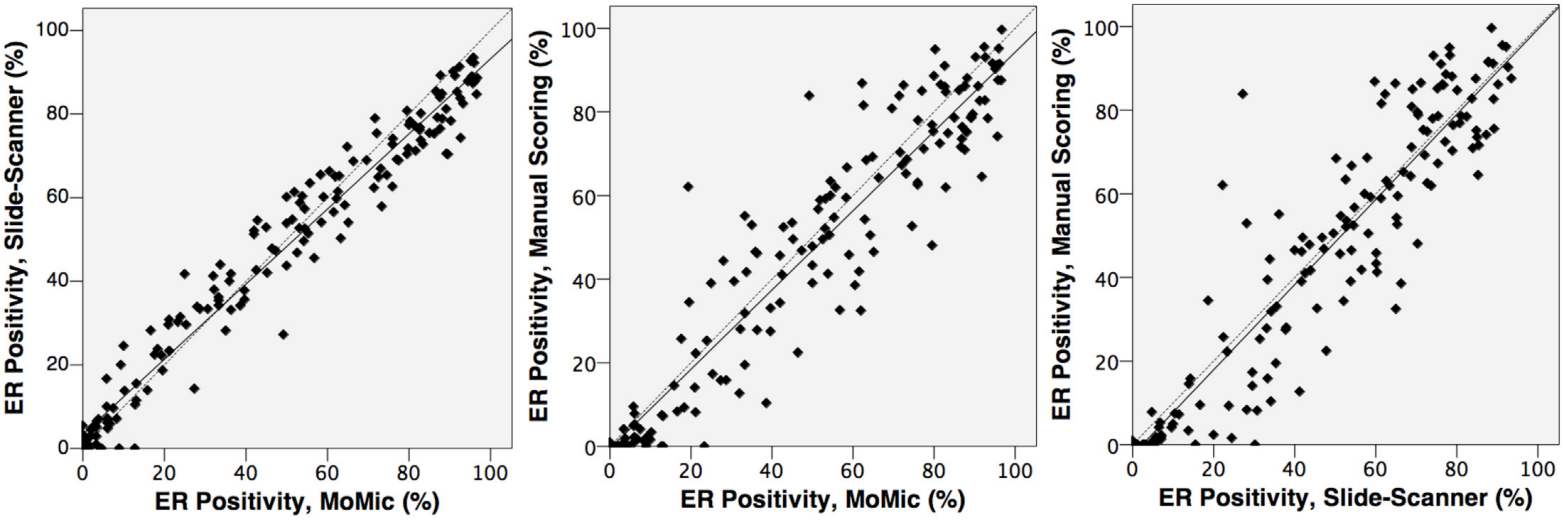
Estrogen receptor status (percentage of cell nuclei staining positively for ER expression), as assessed by the different methods. From left to right: 1) Detected ER positivity by image-analysis of images from point-of-care microscope, compared to images from the reference slide-scanner. 2) Detected ER positivity by image-analysis of images from point-of-care microscope, compared to manual scoring. 3) Detected ER positivity by image-analysis of images from reference slide-scanner, compared to images from the point-of-care microscope.

**Fig 6:**
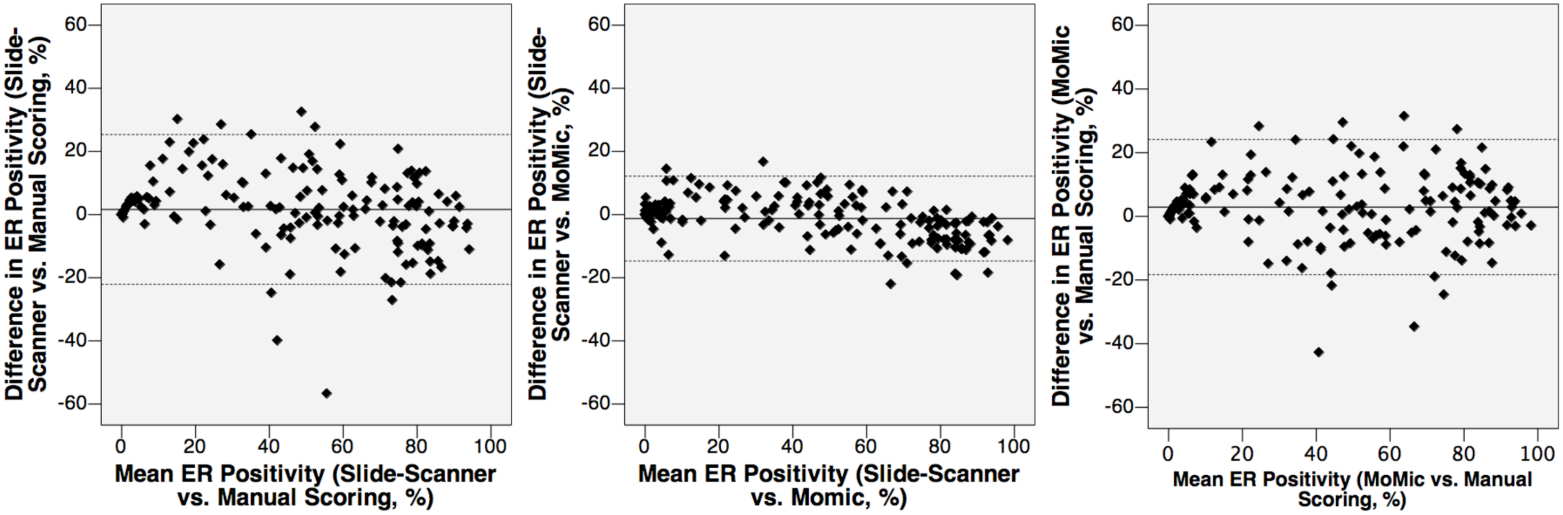
Results from digital image-analysis of sample expressing high levels of estrogen receptors. Left column showing results of ImmunoRatio2-analysis of sample digitized with point-of-care microscope. Middle column showing analysis of reference slide-scanner image. Right column showing magnifications of images, as indicated by red bounding boxes. Positive nuclei marked by blue dots, negative nuclei marked by yellow dots. This particular sample was visually scored as strongly ER positive (82%).

The total number of samples analyzed was 170 after exclusion of samples used for software configuration (n = 10) and samples where matching of the tumor regions was not possible (n = 13). By visual scoring, 33 (19%), 18 (11%) and 119 (70%) breast cancers were classified as ER negative, weakly positive and positive, respectively (Table 1 and S2 Table). Median percentage of positively staining nuclei in the manually scored samples considered as ER positive was 55%. A strong correlation in the ER scores was obtained between the two devices (r = 0.98, p < 001), between the point-of-care microscope and the manual scoring (r = 0.94, p < 0.001) and between the whole-slide scanner and the manual scoring (r = 0.93, p < 0.001; Fig 5). Overall, the interobserver agreement between the different methods of ER assessment was good, and particularly high between the point-of-care microscope and the whole-slide scanner (kappa = 0.84, CI95% = 0.74–0.93, Fig 6).

**Table 1:** Distribution of the classification results of the estrogen receptor-status in the breast cancer samples.

ER Score	Manual Scoring	Slide-Scanner	MoMic
**Negative (<1%)**	33	19	17
**Weak (1–10%)**	18	21	25
**Strong (>10%)**	119	130	128
**Total**	170	170	170

Results from estrogen receptor-assessment, as detected by the three methods, and classified into three categories: ER negative (<1% positivity), weakly ER positive (1–10% positivity) and strongly ER positive (>10% positivity).

**Fig 7:**
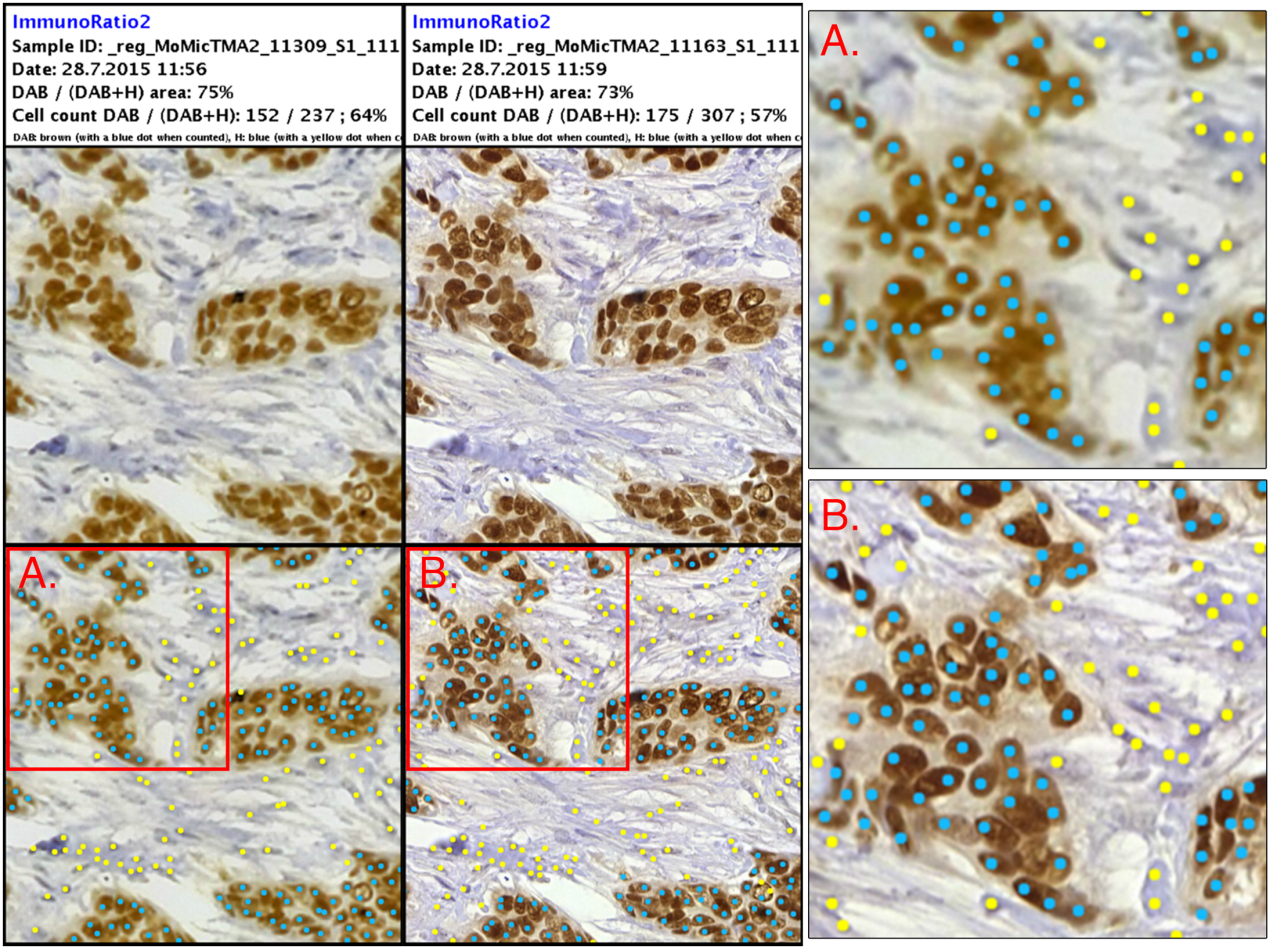
Agreement between the different methods of estrogen receptor-assessment, depicted with Bland-Altman diagrams. From left to right: 1) Mean value of detected estrogen receptor expression by manual scoring and analysis of slide-scanner images, plotted against difference in detected positivity. 2) Mean value of detected estrogen receptor expression by image-analysis of point-of-care microscope images and of slide-scanner images, plotted against difference in detected positivity. The highest concordance was detected between the two device-based scorings. 3) Mean value of detected estrogen receptor expression by image-analysis of point-of-care microscope images and manual scoring, plotted against difference in detected positivity.

When tumor classification as either ER positive or negative was considered, 14 (8%) tumor samples were differently classified when comparing visual scoring to the slide-scanner, and 16 (9%) cases when visual scoring was compared with the point-of-care microscope (Table 2). These cases were considered false positives, as all cases represented samples classified as ER positive by one or both of the devices, and as ER negative by visual scoring. No false negatives were detected with either device. The discrepant cases were visualized as outliers when the logarithm of the percentage of ER positive nuclei detected with either device was plotted against the logarithm of the positivity from the manual scoring (S1 Fig). The concordance in the percentages of positive tumor cell nuclei is depicted as Bland-Altman diagrams in Fig 7. No significant difference in the results from the scoring was observed when the results obtained from the two clinical series were compared separately. When independently examined, the distribution of ER expression (as measured by manual assessment) was similar, although the FinProg samples had an overall slightly higher mean level of ER expression (S2 Fig).

**Table 2:** 

	Reference Slide Scanner			
**MoMic**	<1%	1–10%	>10%	**Total**
<1%	14	3	0	17
1–10%	4	18	3	25
>10%	1	0	127	128
**Total**	19	21	130	170

Results from the assessment of estrogen receptor expression by digital image-analysis of images from reference slide-scanner and the point-of-care microscope. ER status was classified into three groups; ER-negative (<1% positive nuclei), weakly ER positive (1–10% positive nuclei) and strongly positive (>10% positive nuclei).

## Discussion

**Fig 8:**
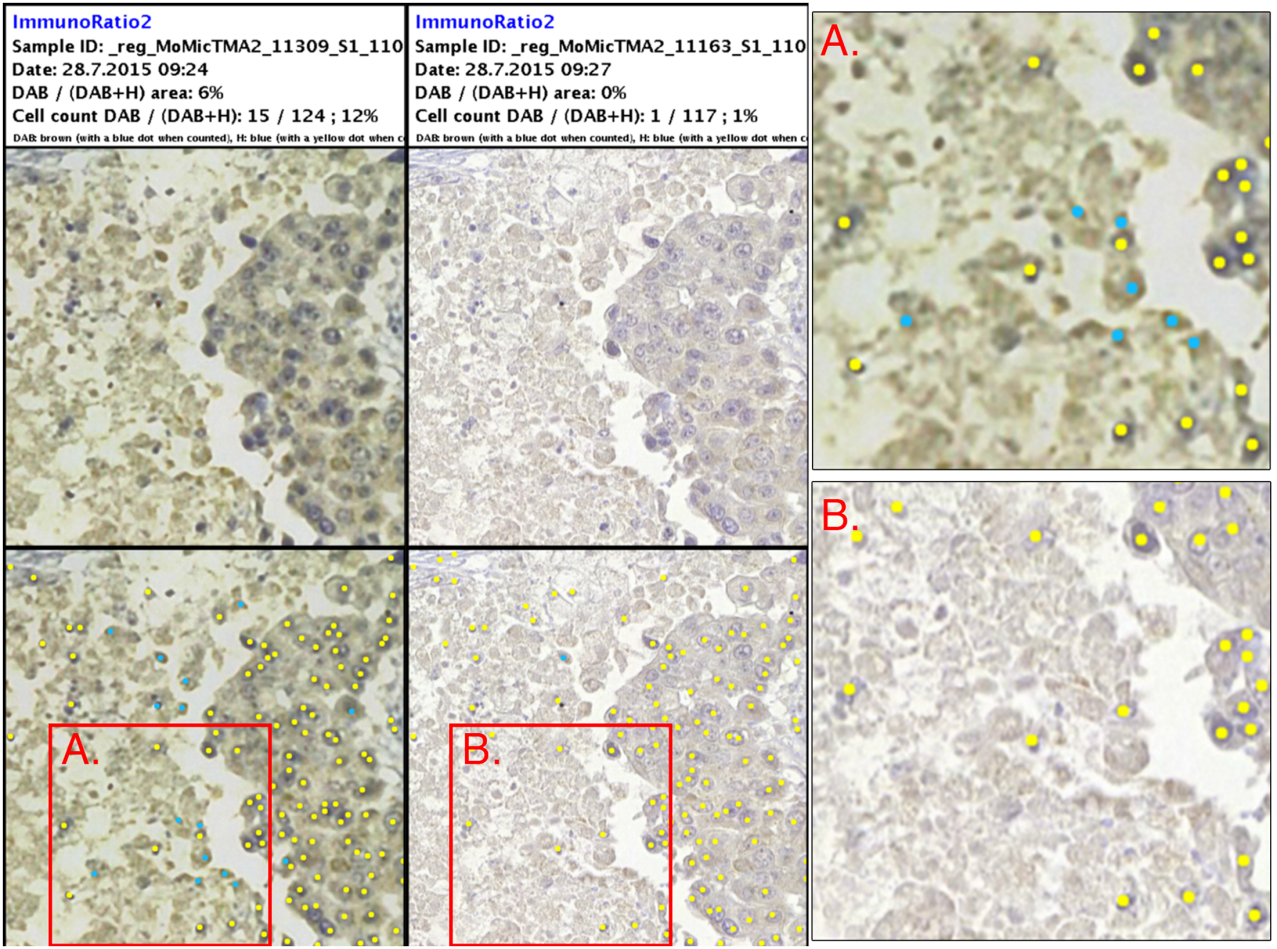
Results from digital image-analysis of borderline sample. Left column showing results of ImmunoRatio2-analysis of sample digitized with point-of-care microscope. Middle column showing analysis of reference slide-scanner image. Right column showing magnifications of images, as indicated by red bounding boxes. Positive nuclei marked by blue dots, negative nuclei marked by yellow dots. Sample scored as ER negative (0%) by visual scoring.

Overall there was a substantial agreement and correlation between the different methods of ER assessment. The results from both imaging devices correlated well with the manual scoring, with a substantial interobserver agreement. More importantly, the agreement between the devices was very strong as defined by the result of the Kappa test (S1 Table). As expected, the weakly positive samples (1–10% ER positivity) proved to be the most challenging to analyze, yielding results differing most from the manual scoring. Scoring of borderline samples has been observed in other studies to cause significant interobserver variability [7–9]. Important to note here is that the analysis of these cases proved to be challenging regardless of which device was used for the digitization, indicating that the spatial quality of the virtual samples was not the main cause. Despite the strong agreement between the machines, the analysis of the samples from the miniature microscope resulted in overall two more discrepant cases, compared to the reference slide scanner. These samples also represented weakly positive samples. A possible cause for this is the lower spatial resolution of the device, which resulted in a slight blurring of smaller, more heavily stained areas and sample preparation artifacts causing them to be detected as positive nuclei (Fig 8). Incorrect scoring of samples is clinically a significant problem, directly affecting patient treatment and medication. This emphasizes the need for manual validation of results in digital image-analysis of immunohistochemically stained samples for optimal results, especially regarding weakly positive cases.

Low-cost, cloud-connected portable digital microscopy have the potential to significantly contribute to global healthcare in both low and high resource countries. In low-resource settings and rural areas with poor access to laboratory facilities and clinical expertise, the technology could be utilized to alleviate the lack of access to clinical microscopy and healthcare. By combining the imaging-devices with computer-assisted image analysis, which can be performed instantly in the cloud, e.g. utilizing high performance grid computing, the setup could provide a powerful platform for diagnosis in areas lacking clinical expertise and trained medical personnel. As laboratory infrastructure supporting immunohistochemical staining procedures is still not available in many rural areas, a number of other potential clinical applications for the technology should also be mentioned. Potentially this technique could be used not only for biomarker detection in cancer diagnostics, but also in the diagnosis of infectious diseases, e.g. malaria and tuberculosis, which remain a significant burden on society in many resource-limited countries. In developed countries, the problem with inconsistent manual scoring of histological samples and lack of interobserver agreement still remains an issue [16, 27, 28, 29]. Increased accessibility of digital microscopy platforms with image-analysis software could be used to improve standardization in clinical pathology, and save both time and resources by reducing the need for manually performed, routine pathological work.

A limitation of this study is the small tumor regions used for the analysis, which occasionally resulted in images with a relatively low amount of visible cells. This proved to be problematic as, for example, a single staining artifact detected as a positive cell in an otherwise clearly negative sample significantly raised the overall positivity. This most likely also contributed to the detection of false positive cases. As the overall amount of detected nuclei in the samples was lower in the samples digitized with the miniature digital microscope, likely due to the lower spatial resolution, we hypothesize that this might have affected this device more. In clinical pathology practice, significantly larger regions of the samples need to be analyzed to provide more clinically reliable results. In this study we used tissue samples from two different clinical series, which were stained using different antibodies. Although we did not observe any significant differences in the digital analysis, results or distribution of ER-expression values when comparing the two series of samples, different staining protocols could potentially affect the results of both visual scoring and digital image-analysis. To provide clinically accurate and consistent results, both the staining process and the tissue handling would also need to be more rigorously standardized. The similarities in the discrepancies in the results acquired with both devices, compared to the visual scoring, also question the reliability and consistency of the manual scoring, especially of the borderline samples. Reference tissue slides with ground truth annotations performed by experienced pathologists on associated virtual slides would be needed for a more objective evaluation of both imaging devices and associated image analysis algorithms. However, to our knowledge no such slides with cell-level annotations are currently available and should be subject to future joint development projects within the pathology community.

## Conclusion

We have showed that results of computer-assisted quantification of ER expression in samples digitized with a low-cost, portable digital microscope yields results comparable to those obtained with a conventional whole-slide scanner. These findings support the research hypothesis but also highlight the fact that computer-assisted analysis of digital slides still requires the supervision of trained personnel, i.e. experienced pathologists for optimal accuracy. Potential other applications of this technology include analysis of other cancer biomarkers and proteins, and detection of pathogens in infectious diseases. We predict that quantification of cytoplasmic and membranous stains is also achievable with this technology; this however will have to be confirmed by further studies. As this study serves as a proof of concept, future work should focus on further evaluation of the reliability, usability and other possible clinical applications of point-of-care mobile microscopy platforms combined with digital image-analysis software.

## Supporting Information

S1 Fig Logarithmic values of estrogen receptor-expression as assessed by different methods. By plotting the logarithmic values of detected ER positivity, the discrepant cases can be visualized clearer as outliers.
(TIF)

S2 Fig Relative distribution of ER expression, as measured by manual scoring of both series of samples. (TIF)

S3 Fig USAF 1951 Resolution Charts captured with the MoMic prototype and a corresponding high-end system. Pictures of a USAF 1951 Resolution Chart captured with the MoMic prototype (upper images) and a corresponding high-end system (lower images) featuring a 20x objective (Plan-Apochromat; numerical aperture 0.8, Zeiss Microscopy AG, Jena, Germany), an 1.0 camera adapter, an RGB LED illuminator (Tofra RGB LED, Tofra Inc, Palo Alto, California, USA), a microscope camera (Nuance FX with a 2/3″ charge-coupled device sensor, Sony ICX825 with a pixel size of 6.45 μm, PerkinElmer, Waltham, MA) and software for image capture (Nuance, PerkinElmer, Waltham, MA) show the current resolution differences. We highlight parts of the USAF chart (group 6, element 5) that correspond to an object size equal to the smallest estrogen receptor expressing nuclei (approx. 5 um) and show that at this level the capability of the prototype to resolve the line pairs is comparable to the high-end setup.
(TIF)

S1 Table Interobserver agreement of determination of estrogen receptor expression. Crosstabulation of agreement in ER assessment, measured between the different methods, pairwise calculated using unweighted kappa statistics.
(DOCX)

S2 Table Excel-spreadsheet with results underlying research findings. (XLSX)
