# Supplementary figures and images for: Quantification of Estrogen Receptor-Alpha Expression in Human Breast Carcinomas With a Miniaturized, Low-Cost Digital Microscope: A Comparison with a High-End Whole Slide-Scanner
Source: PLoS One. 2015 Dec 14;10(12):e0144688. doi: 10.1371/journal.pone.0144688 (PMC4684374; doi:10.1371/journal.pone.0144688)

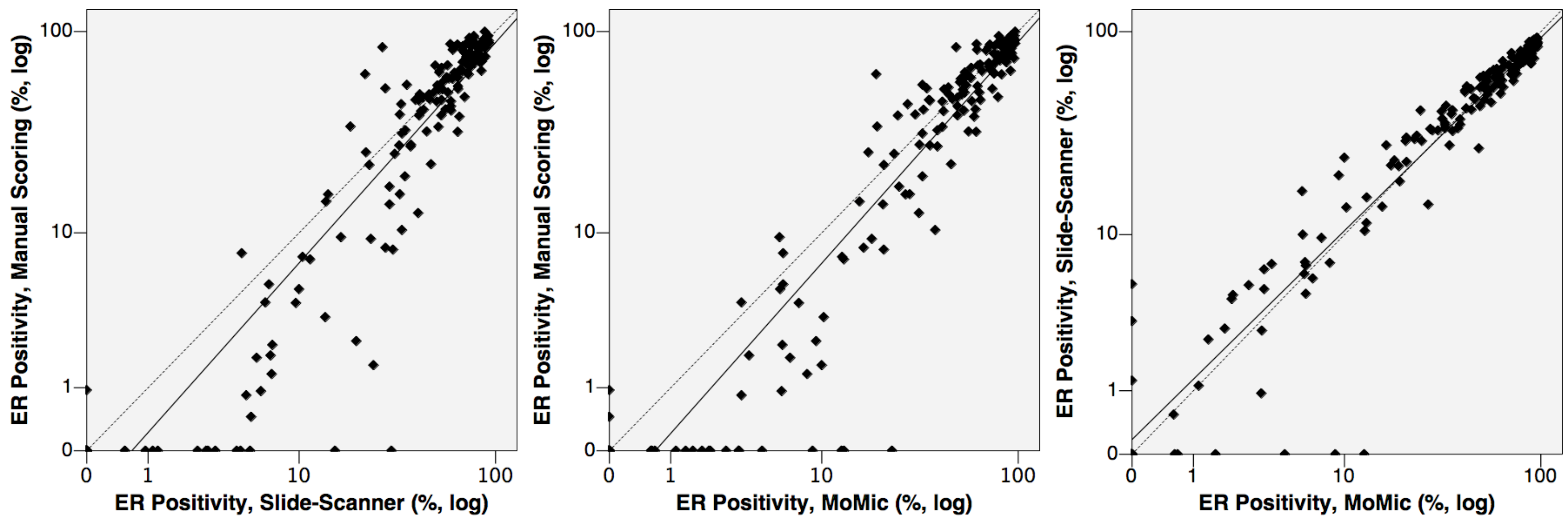

Supplement: S1 Fig — By plotting the logarithmic values of detected ER positivity, the discrepant cases can be visualized clearer as outliers. (TIF) [file pone.0144688.s001.tif]

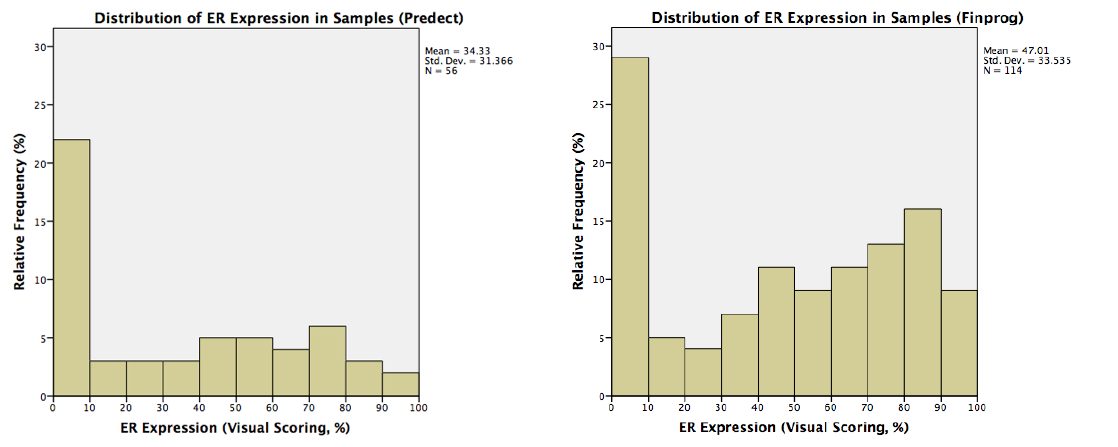

Supplement: S2 Fig — (TIF) [file pone.0144688.s002.tif]
