# Supplementary material for: Quantification of Estrogen Receptor-Alpha Expression in Human Breast Carcinomas With a Miniaturized, Low-Cost Digital Microscope: A Comparison with a High-End Whole Slide-Scanner
Source: PLoS One. 2015 Dec 14;10(12):e0144688. doi: 10.1371/journal.pone.0144688 (PMC4684374; doi:10.1371/journal.pone.0144688)
Supplement: S1 Table — Crosstabulation of agreement in ER assessment, measured between the different methods, pairwise calculated using unweighted kappa statistics. (DOCX) [file pone.0144688.s004.docx]

**S1 Table.** **Interobserver agreement of determination of estrogen receptor expression.** Crosstabulation of agreement in ER assessment, measured between the different methods, pairwise calculated using unweighted kappa statistics.

| **Kappa** | **Manual Scoring** | **Slide-Scanner** | **MoMic** |
| --- | --- | --- | --- |
| **Manual Scoring** | 1 | 0.685 | 0.705 |
| **Slide-Scanner** | 0.685 | 1 | 0.836 |
| **MoMic** | 0.705 | 0.836 | 1 |
